# Adipose tissue distribution in metabolic disease: depot-specific biology, clinical assessment, and therapeutic remodeling

**DOI:** 10.3389/fendo.2026.1822659

**Published:** 2026-06-24

**Authors:** Anke LiuLi, Tongtong Zhang, Zhonghui Feng

**Affiliations:** 1 School of Life Science and Engineering, Southwest Jiaotong University, Chengdu, Sichuan, China; 2 Obesity and Metabolism Medicine-Engineering Integration Laboratory, Department of General Surgery, The Third People’s Hospital of Chengdu, Chengdu, Sichuan, China

**Keywords:** adipose tissue distribution, brown adipose tissue, ectopic fat, fat redistribution, metabolic disease, subcutaneous adipose tissue, visceral adipose tissue

## Abstract

Adipose tissue distribution is a key determinant of metabolic health and cannot be fully explained by total fat mass or body mass index alone. Major adipose compartments, including subcutaneous adipose tissue (SAT), visceral adipose tissue (VAT), thermogenic brown and beige adipose tissues, and ectopic lipid accumulation in non-adipose organs, differ markedly in cellular composition, developmental origin, endocrine function, inflammatory status, and metabolic activity. Excess visceral and ectopic fat is strongly associated with insulin resistance, type 2 diabetes mellitus (T2DM), cardiovascular disease (CVD), metabolic syndrome (MetS), and organ-specific metabolic injury, whereas lower-body SAT may provide a relatively safe lipid-buffering reservoir. Advances in imaging, body-composition analysis, functional metabolic assessment, single-cell omics, and genetic studies have refined the characterization of adipose tissue distribution and its regulatory basis. Current evidence indicates that regional fat patterning is governed by integrated adipogenic, thermogenic, developmental, endocrine, genetic, and environmental programs, and is further modified by sex, age, ethnicity, diet, physical activity, sleep, and stress. Therapeutic strategies also differ substantially in their effects on adipose distribution. Lifestyle intervention and glucagon-like peptide-1 receptor agonists mainly reduce visceral and ectopic fat alongside overall weight loss, thiazolidinediones (TZDs) more directly reshape lipid partitioning, and thermogenic approaches such as cold exposure and β3-adrenergic stimulation remain biologically attractive but not yet established for routine clinical use. Rather than treating adipose depots as isolated anatomical compartments, this review proposes an integrated framework linking depot-specific biology, lipid-buffering capacity, clinical assessment, and therapeutic remodeling. This framework highlights the need for standardized depot-specific phenotyping and precision therapies that target metabolically harmful fat distribution rather than total adiposity alone.

## 1 Introduction

Obesity is a chronic metabolic disease characterized not only by excessive fat accumulation, but also by profound changes in adipose tissue function, cellular composition, and regional fat patterning (1–3). Traditionally, obesity has been defined and classified mainly by body mass index (BMI). Although BMI remains useful for population-level screening, it provides limited information on fat location, tissue quality, or metabolic risk (4–6). Individuals with similar BMI values may show markedly different patterns of visceral fat expansion, subcutaneous fat storage, thermogenic fat activity, and ectopic lipid deposition, leading to substantial differences in cardiometabolic vulnerability (7). Therefore, understanding adipose tissue distribution has become essential for explaining why obesity-related risk varies so widely among individuals.

Adipose tissue is no longer regarded as a passive energy reservoir. It is a metabolically active and immunoendocrine organ composed of mature adipocytes, adipocyte progenitors, immune cells, endothelial cells, fibroblasts, extracellular matrix components, and depot-specific stromal populations (8–10). These cellular and structural features differ across anatomical sites and give rise to distinct biological functions (11). Subcutaneous adipose tissue (SAT), particularly gluteofemoral SAT, often functions as a relatively safe lipid-buffering compartment and may protect against lipid spillover (12). By contrast, visceral adipose tissue (VAT) is more strongly associated with increased free fatty acid flux, chronic low-grade inflammation, adipokine dysregulation, insulin resistance, type 2 diabetes mellitus (T2DM), cardiovascular disease (CVD), and metabolic syndrome (MetS) (2). Beyond classical SAT and VAT, brown and beige adipose tissues contribute to thermogenesis and substrate utilization (13), whereas ectopic lipid accumulation in the liver, skeletal muscle, pancreas, heart, and kidney represents a key pathological consequence of impaired adipose storage capacity (14, 15).

Recent progress in imaging and body-composition assessment has greatly improved the ability to quantify regional adipose depots (16, 17). Anthropometric indices such as waist circumference (WC) and waist-to-hip ratio remain practical screening tools, whereas computed tomography, magnetic resonance imaging, dual-energy X-ray absorptiometry, and magnetic resonance spectroscopy provide more direct characterization of visceral, subcutaneous, and organ-specific fat accumulation (18, 19). In parallel, functional approaches, including stable isotope tracer studies, arteriovenous balance methods, and microdialysis, allow adipose tissue distribution to be evaluated not only anatomically but also metabolically. Together, these methods support a shift from crude obesity classification toward depot-specific and function-oriented adipose phenotyping (20, 21).

The determinants of adipose tissue distribution are complex and multilayered. At the molecular level, adipogenic transcriptional networks centered on peroxisome proliferator-activated receptor γ and CCAAT/enhancer-binding proteins regulate adipocyte differentiation and lipid storage capacity (22, 23). Thermogenic and developmental regulators, including PR domain containing 16 (*PRDM16*), peroxisome proliferator-activated receptor gamma coactivator 1α/β, *TBX15*, *RSPO3*, *GPC4*, *FAM13A*, and WNT/β-catenin signaling, further shape depot identity and thermogenic potential (24–26). These intrinsic programs interact with sex hormones, aging, ethnicity, genetic susceptibility, diet, physical activity, sleep, circadian rhythm, and psychosocial stress. As a result, adipose tissue distribution is a dynamic phenotype rather than a fixed anatomical trait (27, 28).

Because adipose tissue distribution is modifiable, it has become an important target for disease prevention and therapeutic intervention (29). Lifestyle strategies, especially dietary optimization and exercise, remain the most practical first-line approaches for reducing visceral and ectopic fat. Pharmacological interventions show more heterogeneous effects: metformin mainly improves metabolic function with modest effects on adiposity (30), glucagon-like peptide-1 receptor agonists reduce central and hepatic fat largely in parallel with weight loss, thiazolidinediones (TZDs) more directly alter lipid partitioning toward subcutaneous storage, and emerging thermogenic or neuroendocrine strategies remain incompletely validated (31, 32).

Rather than cataloguing adipose depots, this review frames adipose tissue distribution as a dynamic balance between safe lipid storage, depot dysfunction, lipid spillover, and therapeutic remodeling. By connecting depot-specific biology with clinical assessment and intervention strategies, we distinguish anatomical fat accumulation from metabolically harmful adiposity, separate causal evidence from associative findings, and discuss how depot-focused phenotyping may support more precise risk stratification and obesity treatment.

### 1.1 Literature search strategy

This review was conducted as a narrative review. A structured literature search was performed in PubMed, Web of Science, Embase, and Google Scholar from database inception to May 2026. Search terms included combinations of “adipose tissue distribution,” “body fat distribution,” “depot-specific adiposity,” “subcutaneous adipose tissue,” “visceral adipose tissue,” “ectopic fat,” “brown adipose tissue,” “beige adipocytes,” “computed tomography,” “magnetic resonance imaging,” “dual-energy X-ray absorptiometry,” “adipose tissue metabolism,” “genetics,” “sex differences,” “ethnicity,” “diet,” “exercise,” “GLP-1 receptor agonists,” “thiazolidinediones,” “cold exposure,” and “β3-adrenergic agonists.” Original studies, systematic reviews, meta-analyses, clinical guidelines, consensus statements, and relevant high-quality reviews were included when they addressed adipose depot biology, assessment methods, disease associations, regulatory mechanisms, or intervention strategies. Preclinical studies were considered when they provided mechanistic insight into adipose tissue distribution or depot-specific remodeling. Articles with limited relevance, insufficient methodological detail, or no direct connection to adipose tissue distribution were excluded. Given the narrative design, no formal meta-analysis or risk-of-bias assessment was performed; evidence was synthesized thematically according to the main sections of the review.

### 1.2 Conceptual framework and added value of this review

Adipose tissue distribution should be viewed not simply as a map of where fat is stored, but as a readout of how safely the body handles surplus energy. In this view, regional adiposity reflects the balance among lipid-buffering capacity, depot expandability, lipid spillover, and pathological fat accumulation in visceral and ectopic sites. SAT, especially lower-body SAT, can act as a relatively safe metabolic sink when its expansion is supported by effective adipogenesis, vascular adaptation, and controlled extracellular matrix remodeling. Once this buffering capacity is overwhelmed or becomes dysfunctional, excess lipids are more likely to accumulate in VAT and non-adipose organs, including the liver, skeletal muscle, pancreas, heart, and kidney. This shift from adaptive storage to lipid spillover provides a mechanistic bridge between adipose tissue distribution and metabolic disease (3, 15, 29).

Building on this concept, this review treats adipose tissue distribution as a dynamic biological and clinical continuum rather than a fixed classification of fat depots. At one end of this continuum, preserved SAT expandability supports lipid buffering and metabolic flexibility. At the other end, VAT expansion, ectopic lipid deposition, adipose inflammation, fibrosis, and impaired thermogenic capacity contribute to insulin resistance, T2DM, metabolic dysfunction–associated steatotic liver disease, CVD, and other obesity-related complications. Thus, the metabolic risk of obesity depends not only on total fat mass, but also on whether adipose tissue can store energy safely, which depots become dysfunctional, and whether lipid excess has spilled over into organs not designed for major lipid storage (2, 12, 15).

The main value of this review is to connect three areas that are often discussed separately: depot-specific adipose biology, clinical assessment of fat distribution, and therapeutic remodeling of regional adiposity. We first summarize how SAT, VAT, thermogenic adipose tissue, and ectopic lipid deposition differ in cellular composition, developmental origin, endocrine and inflammatory activity, lipid-handling capacity, and disease relevance. We then link these biological differences to clinical phenotyping, emphasizing that BMI and simple anthropometric indices remain useful for screening but cannot define metabolically harmful adiposity on their own. Imaging-based and functional methods, including CT, MRI, DXA, MRI-PDFF, stable isotope tracer studies, arteriovenous balance approaches, and microdialysis, provide complementary information on adipose anatomy and metabolic activity. Finally, we evaluate whether current interventions truly remodel fat distribution or mainly reduce overall adiposity, because weight loss, VAT reduction, ectopic fat reduction, lipid repartitioning, and thermogenic activation are related but not interchangeable outcomes (16, 18, 20, 21).

A further aim of this review is to apply a more critical evidence-based lens. Throughout the manuscript, we distinguish human evidence from preclinical findings, especially when discussing depot-specific mechanisms, thermogenic adipose biology, and pharmacological browning strategies. We also separate associative findings from causal evidence. For example, the link between VAT or ectopic fat and cardiometabolic disease is strongly supported by human imaging and epidemiological studies, whereas many mechanistic explanations still rely heavily on experimental models (2, 33). Likewise, BAT activation and beige adipocyte recruitment remain attractive therapeutic concepts, but their long-term clinical impact on durable fat redistribution is still uncertain (13, 31, 32). By making these distinctions explicit, this review avoids treating all depot-related observations as equally established.

Overall, we propose a shift from a fat-mass-centered model toward a depot-function-centered model of obesity-related risk. This framework integrates regional adipose anatomy, adipose tissue function, ectopic lipid burden, genetic and endocrine background, lifestyle exposure, and therapeutic response. It may help refine risk stratification, identify metabolically high-risk individuals who are missed by BMI-based classification, and guide interventions that target harmful fat distribution rather than body weight alone.

## 2 Biological basis of depot-specific fat distribution

### 2.1 Cellular and functional heterogeneity of adipose tissue

Adipose tissue is a metabolically active and structurally heterogeneous organ composed of mature adipocytes, preadipocytes, immune cells, endothelial cells, fibroblasts, extracellular matrix components, and depot-specific stromal populations (8, 9). This cellular diversity allows adipose tissue to act not only as a lipid reservoir, but also as an endocrine, immune, vascular, and local regulatory organ. Recent single-cell studies have further shown that human adipocytes are not uniform. They include classical adipocytes mainly involved in lipolysis, lipogenesis, and energy storage, as well as nonclassical adipocytes enriched in genes related to angiogenesis, immune regulation, and extracellular matrix remodeling (10). This heterogeneity provides an important biological basis for why different adipose depots vary in lipid-handling capacity, inflammatory tone, endocrine output, and metabolic risk.

Functionally, adipose tissue coordinates whole-body energy homeostasis by storing excess glucose and free fatty acids as triglycerides after feeding and releasing fatty acids through lipolysis during fasting or exercise (34, 35). Brown and beige adipocytes add a thermogenic dimension to this system by dissipating energy through uncoupling protein 1 (*UCP1*)-mediated nonshivering thermogenesis (36, 37). Beyond lipid storage and mobilization, adipose tissue regulates insulin-stimulated glucose uptake, adipokine secretion, lactate production, and systemic glucose and lipid metabolism (8, 38–40). It is also a major immunometabolic organ. In lean states, immune cells such as M2-like macrophages help maintain tissue homeostasis, whereas obesity-driven adipocyte hypertrophy promotes chemokine release, macrophage infiltration, and increased production of inflammatory mediators such as tumor necrosis factor-α (TNF-α) and interleukin-6 (IL-6) (41, 42). This adipocyte–immune cell crosstalk can impair insulin sensitivity, disrupt lipolytic control, and amplify systemic inflammation (43–46).

Importantly, these structural and functional properties are not identical across adipose depots. Subcutaneous, visceral, thermogenic, and ectopic fat compartments differ in cellular composition, vascularization, innervation, extracellular matrix organization, and inflammatory status. Adipose tissue heterogeneity should therefore be viewed as the biological basis of depot-specific fat distribution, rather than as a general background feature of adipose biology.

### 2.2 Adipose expandability, dysfunction, and lipid spillover

The metabolic impact of adipose expansion depends not only on the amount of fat gained, but also on the way adipose tissue expands. Under metabolically favorable conditions, expansion occurs mainly through adipocyte hyperplasia, in which precursor cells proliferate and differentiate into new, relatively small adipocytes with preserved insulin sensitivity and lipid-storage capacity (3, 47–49). This adaptive growth requires coordinated adipogenesis, sufficient vascularization, flexible extracellular matrix remodeling, and controlled immune adaptation. In this state, especially within expandable SAT, surplus energy can be stored relatively safely, reducing lipid exposure to visceral and ectopic sites.

When chronic energy excess exceeds this buffering capacity, adipose tissue growth shifts toward maladaptive adipocyte hypertrophy. Enlarged adipocytes are more vulnerable to hypoxia, mechanical stress, impaired insulin action, and dysregulated lipolysis (50–57). These changes promote chemokine release, macrophage recruitment, inflammatory cytokine production, extracellular matrix deposition, and fibrosis (41, 42, 51–53). As remodeling progresses, adipose tissue gradually loses its ability to contain surplus lipids. Excess fatty acids may then spill over into VAT and non-adipose organs, including the liver, skeletal muscle, pancreas, heart, and kidney. This process links adipose dysfunction to ectopic lipid deposition, insulin resistance, and systemic metabolic disease.

The balance between adaptive expansion and pathological remodeling is shaped by both transcriptional and local microenvironmental programs. The classical adipogenic network is centered on peroxisome proliferator-activated receptor γ (PPARγ) and CCAAT/enhancer-binding protein α (C/EBPα), which cooperate to establish mature adipocyte identity and lipid-storage capacity (22, 58–60). Early activation of C/EBPβ and C/EBPδ initiates adipocyte differentiation, while sterol regulatory element-binding protein 1/1c (SREBP1/1c) connects adipogenesis with lipogenesis by reinforcing PPARγ activity and lipid synthesis (61–63). These pathways help determine whether adipose tissue can generate new functional adipocytes or is forced to expand mainly through hypertrophic enlargement.

The local tissue environment is just as important. Adequate angiogenesis supports oxygen and nutrient supply during adipose expansion, whereas insufficient vascular adaptation aggravates hypoxia and inflammation (50, 64–66). Extracellular matrix (ECM) components such as collagens and fibronectin are needed to maintain tissue structure, but excessive or rigid matrix deposition promotes fibrosis, restricts further expansion, and worsens insulin resistance (54, 67–70). Thus, the key question is not simply whether adipose tissue expands, but whether it expands through metabolically safe hyperplasia or maladaptive hypertrophy. Impaired adipose expandability, particularly in subcutaneous depots, provides a mechanistic bridge between energy excess, visceral fat expansion, ectopic lipid deposition, and metabolic disease.

## 3 Depot-specific adipose biology: from safe storage to pathological spillover

The biological relevance of adipose tissue distribution lies not simply in the anatomical location of fat, but in the distinct capacity of each depot to store lipids safely, adapt to metabolic stress, or contribute to pathological lipid spillover. Adipose depots differ in developmental origin, cellular composition, vascularization, innervation, endocrine output, immune status, and metabolic function. Although the classical division into subcutaneous adipose tissue (SAT) and visceral adipose tissue (VAT) remains useful, it is no longer sufficient to explain the complexity of human fat distribution. A more informative framework should include anatomically distinct white adipose depots, thermogenic brown and beige adipose tissues, and ectopic lipid accumulation in non-adipose organs. Within this framework, expandable SAT may serve as a relatively safe lipid-buffering reservoir, whereas excessive VAT expansion and ectopic lipid deposition often signal impaired storage capacity and higher metabolic risk. These depots should therefore be viewed not as isolated fat stores, but as interconnected components of a system that shapes energy homeostasis, immunoinflammatory activity, and organ-specific metabolic injury (3, 7, 71, 72).

### 3.1 White adipose tissue

White adipose tissue (WAT) is the major energy-storage compartment in humans and a key site where the balance between safe lipid storage and pathological lipid spillover is determined. Although WAT is composed mainly of unilocular adipocytes, its biological effects differ substantially across anatomical depots. It has traditionally been classified into subcutaneous adipose tissue (SAT) and visceral adipose tissue (VAT). In general, lower-body SAT is more closely associated with lipid buffering and metabolically safer storage, whereas intra-abdominal VAT is more strongly linked to insulin resistance, dysregulated lipid metabolism, inflammation, and cardiovascular risk (11, 15, 73).

This SAT/VAT distinction is useful, but it should not be treated as fixed or absolute. The metabolic impact of WAT depends on depot location, mode of expansion, vascular and immune remodeling, extracellular matrix dynamics, and whether lipid-storage capacity has been exceeded. Expandable SAT may protect against lipid spillover, whereas maladaptive SAT expansion and excessive VAT accumulation can both drive metabolic dysfunction. WAT is therefore better understood as a functionally heterogeneous system in which depot-specific storage capacity and pathological remodeling jointly shape metabolic risk (74).

#### 3.1.1 Subcutaneous adipose tissue

Subcutaneous adipose tissue (SAT) is the largest adipose depot in humans. It lies beneath the dermis and above the deep fascia, with major deposits in the abdominal, gluteofemoral, and limb regions. Beyond storing and mobilizing triglycerides according to energy demand, SAT also acts as an endocrine organ through the secretion of adipokines such as leptin (11, 75). Compared with VAT, SAT generally has a lower inflammatory tone, greater lipid-buffering capacity, and a more favorable metabolic profile (76–78). It also contributes to mechanical protection and thermal homeostasis (79).

SAT, however, is not metabolically uniform. Human studies support a clear regional distinction: gluteofemoral SAT is often associated with lower cardiometabolic risk, whereas upper-body SAT, especially abdominal SAT, shows higher lipid turnover and lipolytic activity (12, 80, 81). In this sense, lower-body SAT may serve as a longer-term and relatively safer lipid-storage compartment, while abdominal SAT is more metabolically dynamic. Still, SAT should not be viewed as uniformly protective. When its expansion becomes maladaptive, excessive lipid accumulation, adipocyte hypertrophy, inflammation, and reduced expandability may promote lipid spillover and metabolic dysfunction (82).

Preclinical studies have clarified several mechanisms of SAT biology, including adipogenesis, browning potential, immune remodeling, and depot plasticity. Yet their translation to humans needs caution. Human SAT shows more obvious regional partitioning, especially between abdominal and gluteofemoral depots, whereas mouse studies most often examine inguinal white adipose tissue (iWAT) and dermal-associated fat layers (83). These anatomical and functional differences mean that murine SAT findings are valuable for mechanistic insight, but should not be treated as direct equivalents of human SAT biology.

#### 3.1.2 Visceral adipose tissue and subdepots

Visceral adipose tissue (VAT) is not a single uniform depot, but a group of anatomically and functionally distinct fat depots located within the abdominal cavity and around internal organs. These include omental, mesenteric, retroperitoneal, and perirenal fat, as well as cardiac depots such as epicardial and pericardial fat (8). Compared with SAT, VAT is generally more lipolytically active and more closely linked to pro-inflammatory signaling. It releases free fatty acids and inflammatory mediators, including TNF-α, C-reactive protein (CRP), and IL-6, which can affect hepatic and systemic metabolism through portal and systemic circulation (84–88).

Human studies consistently associate excess VAT with insulin resistance, dyslipidemia, hypertension, CVD, MetS, and other obesity-related complications (88–90). This evidence is particularly strong for central obesity and intra-abdominal fat accumulation. Even so, VAT biology remains depot-specific. Omental and mesenteric fat are closely related to portal metabolic flux and immune surveillance, whereas perirenal and cardiac fat may exert more direct local effects on renal and cardiovascular regulation (91–96). VAT should therefore not be treated as a homogeneous compartment in either mechanistic research or clinical risk assessment.

Preclinical models have been useful for clarifying how VAT dysfunction contributes to metabolic disease, including adipocyte hypertrophy, enhanced lipolysis, macrophage infiltration, inflammatory activation, and impaired insulin signaling (97). However, animal-to-human translation is not straightforward. Human VAT is composed mainly of omental and mesenteric depots, whereas many murine studies rely on perigonadal fat, which has no clear human anatomical counterpart (3, 83). Single-cell analyses have also revealed both shared and species-specific features across human and mouse VAT (33). These differences should be kept in mind when using rodent VAT data to interpret human metabolic disease.

Importantly, VAT is not intrinsically harmful under all conditions. Physiologically, it contributes to immune surveillance, mechanical support of visceral organs, and adaptive energy storage (93, 98, 99). Its pathogenic role becomes more prominent when expansion is excessive and accompanied by adipocyte hypertrophy, dysregulated lipolysis, inflammation, and local or systemic metabolic stress. In this sense, the biological impact of VAT depends on the specific subdepot, expansion state, and clinical context.

### 3.2 Thermogenic adipose depots: energy dissipation and metabolic adaptation

Beyond WAT, which mainly stores excess energy, humans also possess thermogenic adipose tissues that dissipate energy and support metabolic adaptation. These depots mainly include brown adipose tissue (BAT) and inducible beige adipocytes (13, 100). Unlike classical white adipocytes, thermogenic adipocytes are rich in mitochondria and express *UCP1*, allowing them to drive nonshivering thermogenesis and enhance substrate oxidation in response to cold exposure and sympathetic activation (101, 102).

Thermogenic adipose depots are therefore relevant to fat distribution not as simple fat stores, but as functional compartments that influence energy expenditure, glucose and lipid clearance, and the metabolic plasticity of WAT. Emerging evidence also suggests that thermogenic programs may extend beyond heat production. For example, injury-induced SAT browning has been linked to skin repair through NRG4-mediated regulation of macrophage polarization and myofibroblast activation (103). These findings broaden the biological meaning of thermogenic fat, although its clinical value for obesity treatment and durable fat redistribution remains to be firmly established.

#### 3.2.1 Brown adipose tissue

Brown adipose tissue (BAT) is the classical thermogenic adipose depot. In adult humans, it is located mainly in the supraclavicular and deep neck regions, with smaller depots in paravertebral and, less consistently, perirenal areas (104, 105). This anatomical pattern only partly overlaps with that in mice, where interscapular BAT is the dominant and most frequently studied depot (106, 107). Such species differences need to be considered when translating BAT findings from animal models to humans.

BAT is characterized by multilocular lipid droplets, abundant mitochondria, and high expression of *UCP1*, which mediates the dissipation of chemical energy as heat (108–110). In addition to thermogenesis, BAT contributes to glucose and lipid clearance and influences systemic metabolic homeostasis through the secretion of multiple batokines (111–113). Human imaging and metabolic studies generally associate higher BAT activity with a more favorable cardiometabolic profile, whereas reduced BAT abundance or activity has been linked to obesity and metabolic dysfunction (114, 115).

Even so, BAT should not be treated as an established therapeutic solution for obesity. Much of the mechanistic evidence still comes from rodent studies, and human BAT activity is influenced by age, sex, adiposity, season, ambient temperature, and detection methods. Whether BAT activation can drive sustained fat loss or clinically meaningful fat redistribution in humans remains uncertain. BAT is therefore best viewed as an important thermogenic depot and a promising metabolic target, but not yet a validated strategy for durable remodeling of adipose distribution.

#### 3.2.2 Beige adipocytes

Beige adipocytes are inducible thermogenic adipocytes that emerge within white adipose tissue, especially subcutaneous depots, in response to cold exposure, β-adrenergic signaling, exercise, nutritional cues, and other metabolic stimuli (116, 117). At rest, they may resemble white adipocytes, but once activated, they acquire multilocular lipid droplets, increased mitochondrial content, *UCP1* expression, and stronger oxidative capacity. This plasticity makes beige adipocytes particularly relevant to WAT remodeling, as beiging can shift WAT toward a more energy-dissipating and metabolically favorable phenotype (118, 119).

Preclinical studies strongly suggest that beige adipocyte recruitment can increase energy expenditure, improve metabolic phenotypes, and protect against diet-induced metabolic dysfunction. In this context, beiging is better viewed as a marker of favorable adipose remodeling rather than simply an increase in thermogenic cell number. Still, translation to humans remains difficult. In mice, beige adipocytes are most commonly found in inducible subcutaneous depots, particularly anterior and inguinal fat, whereas in humans, thermogenic fat with beige or brite-like features is more often detected in cervical, supraclavicular, and paravertebral regions (100, 120, 121).

Thus, beige adipocyte biology should be interpreted with both interest and caution. It offers a strong mechanistic link between WAT plasticity, energy expenditure, and metabolic improvement, but whether beige fat recruitment can be safely, durably, and clinically meaningfully induced in humans remains unresolved. At present, beige adipocytes represent a promising but still incompletely validated target for adipose tissue remodeling.

### 3.3 Ectopic lipid deposition

When adipose storage capacity is exceeded, surplus lipids may accumulate in organs not designed for major fat storage, including the liver, skeletal muscle, heart, pancreas, and kidneys (122, 123). This process, commonly referred to as ectopic lipid deposition, is not an adipose depot in the classical anatomical sense. Still, it is a critical manifestation of disordered fat distribution and an important mediator of obesity-related organ injury (14).

Ectopic lipid deposition is best understood as a downstream consequence of impaired lipid buffering. In this setting, limited SAT expandability, excessive VAT expansion, increased free fatty acid flux, inflammation, and impaired oxidative capacity can all promote lipid accumulation outside conventional adipose tissue (15). Therefore, assessment of fat distribution should not stop at SAT and VAT. A clinically meaningful evaluation also needs to ask whether lipid excess has spilled over into non-adipose organs, where it may contribute to insulin resistance, tissue dysfunction, and disease progression.

#### 3.3.1 Hepatic lipid deposition

Hepatic lipid deposition is one of the most common forms of ectopic lipid accumulation and represents a major pathological basis of metabolic dysfunction–associated steatotic liver disease. Although it often coexists with increased visceral adiposity, the two should not be regarded as equivalent (124). VAT expansion may promote hepatic lipid accumulation by increasing portal free fatty acid delivery and inflammatory exposure, while persistent hepatic steatosis can further worsen hepatic insulin resistance and disturb glucose and lipid homeostasis (125, 126).

Human studies strongly associate hepatic fat with insulin resistance, metabolic dysfunction, and cardiometabolic risk. Still, the mechanism is not always straightforward, because hepatic lipid deposition is shaped by adipose lipolysis, *de novo* lipogenesis, dietary substrate flux, mitochondrial oxidation, inflammation, and genetic susceptibility. Preclinical models provide stronger causal support: high-fat or high-fat/high-sugar feeding in mice induces hepatic steatosis together with obesity, insulin resistance, and progression toward steatohepatitis (127–129).

Thus, hepatic lipid deposition should not be treated simply as a companion feature of visceral obesity. It is better viewed as an organ-specific endpoint of lipid spillover and metabolic stress, linking adipose tissue dysfunction to liver injury and systemic metabolic disease (14).

#### 3.3.2 Intramyocellular and intermuscular lipid deposition

Skeletal muscle lipid deposition mainly includes intramyocellular lipid accumulation, intermuscular fat infiltration, and fat accumulation around muscle tissue (130–132). Compared with total fat mass, these changes more directly reflect impaired lipid utilization, reduced metabolic flexibility, and altered muscle quality (133, 134). Human studies have linked intramuscular and intermuscular fat to insulin resistance, reduced muscle strength, impaired mobility, and age-related sarcopenic phenotypes (135, 136).

However, muscle lipid accumulation should not be interpreted only by quantity. Its metabolic effect depends on lipid species, mitochondrial oxidative capacity, physical activity status, and the balance between lipid storage and lipid use (5, 133, 134, 137, 138). Intramyocellular lipid may be harmful when accompanied by mitochondrial dysfunction and impaired insulin signaling, but not all muscle lipid accumulation carries the same metabolic risk. This distinction is important when interpreting imaging- or biopsy-based measures of muscle fat.

Preclinical studies further support a mechanistic link between obesity-related muscle lipid deposition and metabolic injury. In high-fat diet-induced obesity models, abnormal lipid accumulation in skeletal muscle is often accompanied by impaired insulin signaling, mitochondrial dysfunction, and reduced oxidative capacity (137–139). Skeletal muscle fat should therefore not be viewed as just another measure of adiposity. It is better understood as a marker of lipid spillover into a tissue that plays a central role in glucose disposal and whole-body metabolic flexibility.

### 3.4 Summary: depot-specific adipose biology and metabolic risk

**Figure 1:**
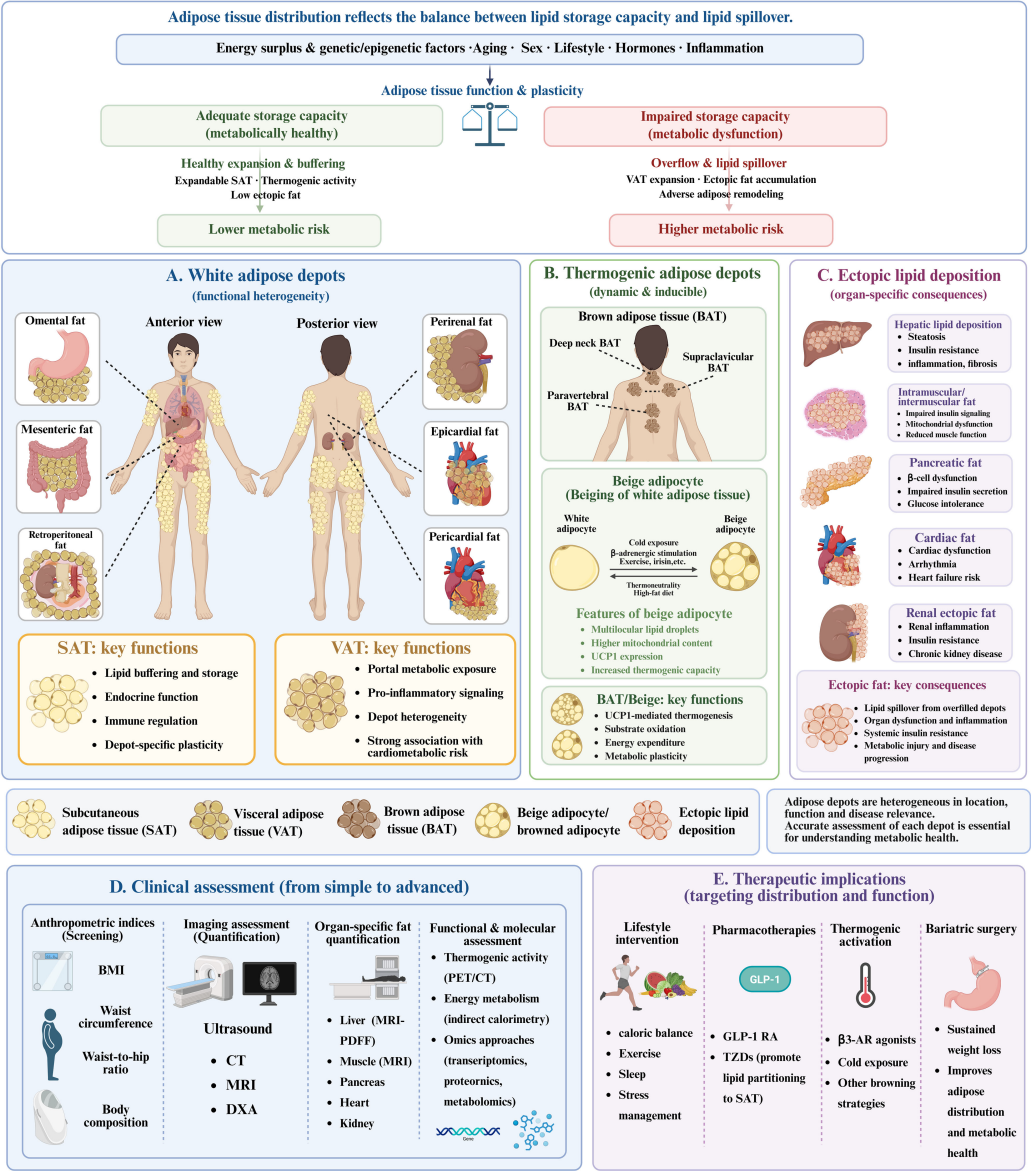
Integrated framework of adipose tissue distribution, lipid-buffering capacity, and metabolic risk. Adipose tissue distribution reflects the balance between lipid-storage capacity and lipid spillover. Energy surplus, together with genetic/epigenetic background, aging, sex, lifestyle, hormonal status, and inflammation, shapes adipose tissue plasticity. When storage capacity is preserved, expandable subcutaneous adipose tissue (SAT) and thermogenic activity support lipid buffering, limit ectopic lipid deposition, and are associated with lower metabolic risk. When storage capacity is impaired, visceral adipose tissue (VAT) expansion, ectopic fat accumulation, and adverse adipose remodeling promote higher metabolic risk. **(A)** White adipose depots are anatomically and functionally heterogeneous. SAT mainly supports lipid buffering, endocrine regulation, immune homeostasis, and depot-specific plasticity, whereas VAT is more closely linked to portal metabolic exposure, pro-inflammatory signaling, depot heterogeneity, and cardiometabolic risk. Representative visceral depots include omental, mesenteric, retroperitoneal, perirenal, epicardial, and pericardial fat. **(B)** Thermogenic adipose depots include brown adipose tissue (BAT) and inducible beige adipocytes. BAT is enriched in the deep neck, supraclavicular, and paravertebral regions, whereas beige adipocytes arise within white adipose tissue in response to cold exposure, β-adrenergic activation, exercise, and dietary cues. These thermogenic cells contribute to uncoupling protein 1 (*UCP1*)-mediated thermogenesis, substrate oxidation, energy expenditure, and metabolic plasticity. **(C)** Ectopic lipid deposition represents lipid spillover into non-adipose organs, including the liver, skeletal muscle, pancreas, heart, and kidney. Unlike classical adipose depots, ectopic fat is closely associated with organ-specific dysfunction, inflammation, insulin resistance, and metabolic disease progression. **(D)** Clinical assessment ranges from anthropometric screening to imaging-based and functional phenotyping, allowing progressive evaluation of overall adiposity, SAT/VAT distribution, organ-specific fat, thermogenic activity, and depot function. **(E)** Therapeutic strategies act through distinct modes, including overall fat loss, lipid repartitioning, metabolic-function improvement, and thermogenic activation. Their depot-specific effects should be interpreted according to weight-loss dependence and evidence for true redistribution.

Taken together, adipose tissue distribution should be understood as a depot-specific metabolic system rather than a simple anatomical map of fat storage. Expandable SAT may buffer surplus lipids relatively safely, whereas excessive VAT expansion and ectopic lipid deposition often reflect impaired storage capacity and greater metabolic risk (3, 12, 15). Thermogenic adipose tissues add a functional dimension by influencing energy expenditure, substrate oxidation, and WAT plasticity, although their clinical relevance for durable fat redistribution remains uncertain (13, 31, 32). This framework separates anatomical fat accumulation from metabolically harmful adiposity and highlights a key translational point: BMI or total fat mass alone cannot capture depot-specific risk. More precise approaches are therefore needed to distinguish SAT, VAT, thermogenic fat activity, and organ-specific lipid deposition, as discussed in the following section (4, 18). **Figure 1** summarizes this framework and links adipose depot heterogeneity to metabolic risk, clinical assessment, and therapeutic implications.

## 4 Methods for assessing adipose tissue distribution

No single method can fully capture all clinically relevant dimensions of adipose tissue distribution. In practice, currently available approaches can be grouped into three broad categories: anthropometric and screening-based measures, imaging and body-composition techniques, and dynamic functional methods that assess regional lipid flux and adipose tissue metabolism (18, 20). Used together, these approaches provide a more complete evaluation of fat distribution, from overall adiposity and regional fat patterning to depot-specific fat accumulation and metabolic activity (19).

### 4.1 Anthropometric and screening-based approaches

Anthropometric measures remain the most accessible first-line tools for assessing adipose tissue distribution in both clinical practice and epidemiological research. These measures mainly include waist circumference (WC), hip circumference (HC), and waist-to-hip ratio (WHR), while body mass index (BMI) continues to be widely used as a basic indicator of overall adiposity (140, 141). Among them, waist circumference (WC) is particularly useful because it reflects abdominal fat accumulation and provides risk information beyond that captured by BMI alone (18, 90). WHR provides a simple index of relative abdominal versus gluteofemoral fat distribution, whereas HC may partly capture the contribution of lower-body adiposity to metabolic risk (142–145). Even so, these indices remain indirect and cannot reliably distinguish SAT from VAT. They should therefore be considered practical screening tools rather than definitive methods for depot-specific phenotyping.

Body mass index (BMI) remains one of the most widely used screening tools for obesity and is calculated as body weight in kilograms divided by height in meters squared (kg/m²). On this basis, individuals can be categorized into different weight-status groups (**Table 1**) (146). Despite its convenience and widespread clinical use, BMI has important limitations. Most notably, it does not distinguish fat mass from lean mass and may therefore misclassify individuals with high muscularity as overweight or obese (147). In addition, BMI provides little information on regional fat distribution, which is a key determinant of metabolic risk (4). Another issue is that BMI cut-offs may vary across populations because body composition and metabolic vulnerability differ by ethnicity (6). For this reason, BMI should be interpreted with caution and, whenever possible, considered alongside other indicators of central adiposity and adipose tissue distribution.

**Table 1:** BMI classification standards.

Classification standard	BMI(Kg/m (^2^))
Underweight	<18.5
Normal weight	18.5-24
Overweight	24-28
Mild obesity	28.0-32.5
Moderate obesity	32.5-37.5
Severe obesity	37.5-50
Morbid obesity	>50

The BMI cut-offs shown in **Table 1** are based on the Chinese Guidelines for the Clinical Management of Obesity (2024 Edition) and are presented for Chinese adults. For international comparison, WHO adult categories define overweight as BMI ≥25 kg/m² and obesity as BMI ≥30 kg/m².

In this review, the BMI classification shown in **Table 1** is based on the Chinese Guidelines for the Clinical Management of Obesity (2024 Edition), which should be interpreted in the context of population-specific criteria.

### 4.2 Imaging and body-composition techniques

Imaging methods address many of the limitations of anthropometric indices by providing direct information on both the amount and anatomical distribution of adipose tissue (16). Computed tomography (CT) and magnetic resonance imaging (MRI) remain the principal reference methods for regional fat quantification (17). CT offers high accuracy for measuring VAT and detecting organ-specific ectopic fat, although its routine use is constrained by exposure to ionizing radiation (148, 149). By contrast, MRI provides high-resolution, radiation-free assessment of SAT, VAT, and organ fat, making it particularly suitable for repeated and longitudinal evaluations (150). In addition, MRI-based proton density fat fraction (PDFF) is now widely used to quantify hepatic fat, while magnetic resonance spectroscopy (MRS) provides complementary chemical information and is useful for assessing hepatic and intramyocellular lipid content (148, 151, 152).

Dual-energy X-ray absorptiometry (DXA) occupies an intermediate position between simple screening tools and advanced imaging modalities (153). It is more accessible and less costly than CT or MRI, provides whole-body and regional body-composition estimates, and can generate indirect estimates of abdominal VAT (154, 155). However, DXA is less precise than CT or MRI for depot-specific fat phenotyping (156). Bioelectrical impedance analysis (BIA), including devices such as InBody, is portable, rapid, and inexpensive, making it useful for routine screening and follow-up (157). Yet BIA provides only indirect estimates, is affected by hydration status and device algorithms, and cannot reliably distinguish SAT from VAT or quantify ectopic fat (16, 158). Overall, CT and MRI are preferred for accurate depot-level characterization, whereas DXA and BIA are more suitable for broader clinical and field-based use.

### 4.3 Dynamic functional assessment of regional adipose metabolism

Static measurements indicate where fat is distributed, but they do not fully capture the metabolic behavior of adipose tissue (159). For mechanistic studies, dynamic functional approaches provide important complementary information. Stable isotope tracer methods can quantify whole-body and regional lipolysis, free fatty acid (FFA) appearance, substrate turnover, and tissue-specific fatty acid uptake (160). When combined with imaging, these approaches can link depot size to lipid flux and metabolic activity (20).

Additional insight can be gained from arteriovenous balance studies performed together with measurements of local adipose tissue blood flow (21). These methods allow direct estimation of regional glycerol and FFA release, helping to define the contribution of specific depots to systemic lipid exposure (161). Microdialysis provides another useful approach for assessing local adipose tissue lipolysis and interstitial metabolite changes *in vivo*, particularly in subcutaneous depots (162, 163). Although these approaches are not part of routine clinical practice, they are highly informative in physiological and translational research because they characterize depot-specific metabolic function rather than fat mass alone (21). Their inclusion highlights that adipose tissue distribution can be evaluated not only anatomically, but also functionally.

Overall, anthropometric indices and BMI are most useful for screening, imaging techniques are essential for precise anatomical quantification, and dynamic functional methods are particularly informative for mechanistic studies of depot-specific metabolism. Thus, the optimal approach should be selected according to the specific clinical or research objective.

**Figure 2:**
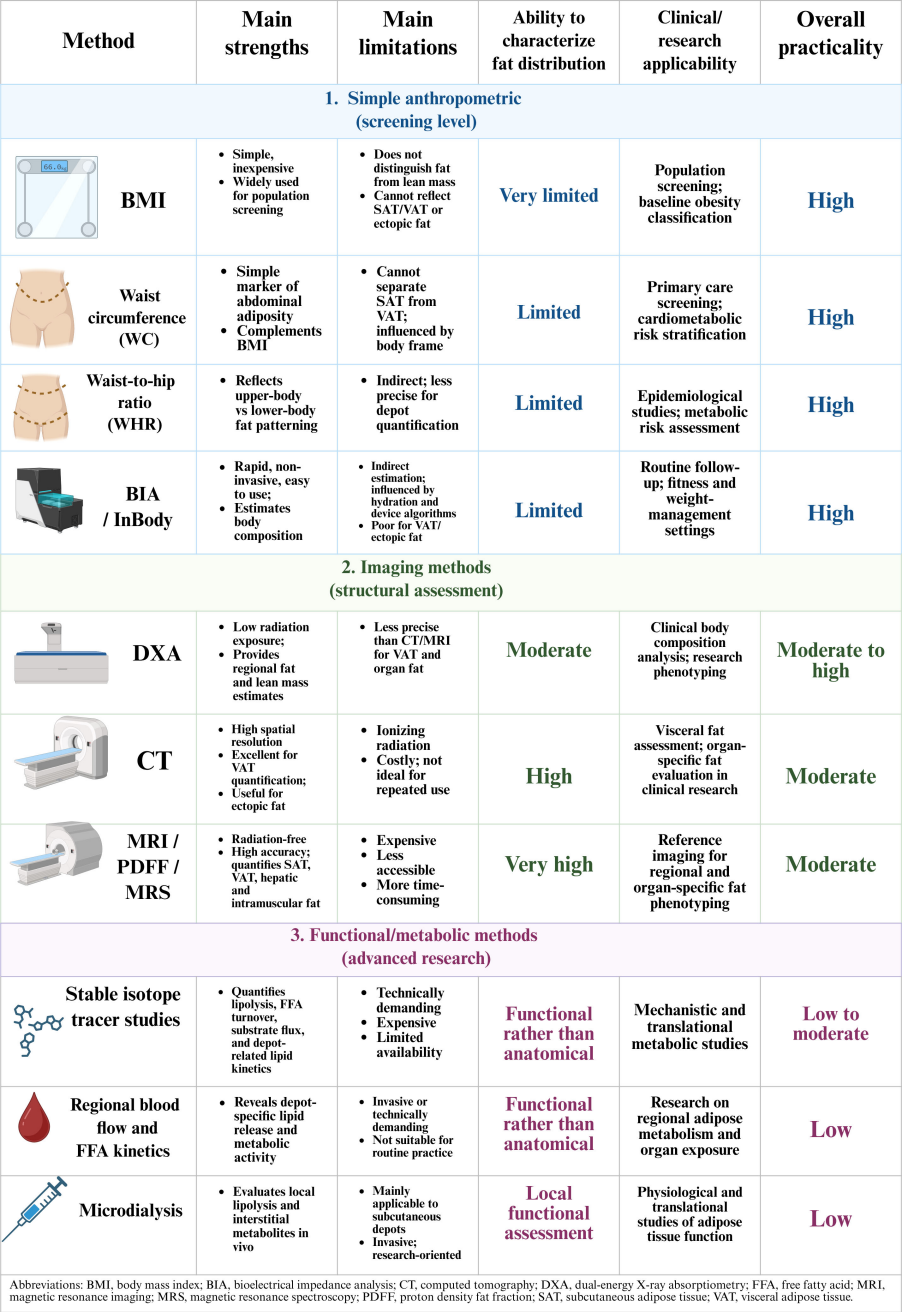
Comparison of methods for assessing adipose tissue distribution and regional adipose function. Anthropometric indices and BMI are most useful for low-cost screening but provide limited information on depot-specific fat distribution. BIA and DXA offer practical estimates of body composition for routine clinical use, whereas CT and MRI-based techniques remain the principal methods for accurate regional and organ-specific fat quantification. Dynamic functional approaches, including stable isotope tracer studies, regional blood flow and FFA kinetics, and microdialysis, are mainly used in mechanistic studies because they assess depot-specific metabolic activity rather than fat mass alone.

A comparison of methods for assessing adipose tissue distribution and regional adipose function is summarized in **Figure 2**.

## 5 Clinical impact and risk stratification of adipose tissue distribution

As methods for assessing adipose tissue distribution have become more precise, its clinical relevance has become increasingly clear. The pathogenic impact of adiposity depends not only on total fat mass, but also on lipid location, depot function, and the extent of lipid spillover into non-adipose organs (164). The strongest and most consistent evidence links excess VAT and ectopic fat to cardiometabolic disease, whereas links with non-classical outcomes, including mental health, remain less mechanistically established (15, 165). Thus, VAT and ectopic fat are better viewed not simply as anatomical descriptors, but as clinically informative features of metabolically harmful adiposity.

### 5.1 Cardiometabolic disease: the strongest clinical evidence

Among adipose depots, VAT shows the strongest and most consistent association with adverse metabolic and cardiovascular outcomes (166). Mechanistically, VAT may contribute to cardiometabolic disease through increased free fatty acid flux, pro-inflammatory cytokine secretion, adipokine dysregulation, and worsening systemic insulin resistance (122, 167, 168). In the cardiovascular system, these processes are linked to endothelial dysfunction, high-risk plaque formation, and atherosclerotic progression, thereby increasing the risk of coronary artery disease and cardiovascular events (88, 169–171).

Human imaging and epidemiological studies provide strong clinical support for this association. Imaging-derived measures of visceral adiposity, as well as population-adapted indices such as the Chinese Visceral Adiposity Index (CVAI), have shown value in cardiovascular risk assessment, particularly in Asian populations (172–175). Cardiac adipose depots, especially epicardial and pericardial fat, have also been associated with heart failure-related hospitalization and all-cause mortality (176–178). These findings suggest that depot-specific adiposity can help refine cardiometabolic risk assessment, especially when BMI alone does not fully capture individual risk.

Nevertheless, the evidence should be interpreted with appropriate caution. Human studies strongly support the association between VAT and cardiovascular risk, but many causal pathways are still inferred from experimental models, mechanistic human studies, or longitudinal associations. VAT is therefore best viewed as both a clinically informative risk feature and a biologically plausible contributor to cardiometabolic disease, rather than a stand-alone causal explanation for all obesity-related cardiovascular complications.

### 5.2 Type 2 diabetes, metabolic syndrome, and ectopic fat-related organ injury

Abnormal adipose distribution is closely linked to type 2 diabetes mellitus (T2DM) and metabolic syndrome (MetS). Excess VAT may promote chronic low-grade inflammation, increase lipid flux, and impair insulin signaling, thereby contributing to systemic insulin resistance (2, 88, 179, 180). Ectopic lipid deposition further strengthens this connection. Hepatic fat is associated with hepatic insulin resistance and disrupted glucose and lipid metabolism, while pancreatic fat may affect β-cell function and insulin secretion (14, 181). Together, VAT and ectopic fat provide a mechanistic bridge between unfavorable fat distribution and metabolic deterioration.

In clinical practice, waist circumference (WC), VAT area, and related adiposity indices remain useful indicators of MetS risk (182–185). Lower-body SAT, particularly gluteofemoral fat, is often associated with a more favorable metabolic profile, whereas limited storage capacity or maladaptive SAT expansion may promote lipid spillover into visceral and ectopic depots (179, 186, 187). This helps explain why individuals with similar BMI can differ markedly in insulin resistance, glycemic status, lipid abnormalities, and overall metabolic risk.

From a translational perspective, depot-specific fat distribution may help identify metabolically high-risk obesity phenotypes. Individuals with central obesity, high VAT burden, hepatic steatosis, pancreatic fat, or skeletal muscle lipid infiltration may need closer metabolic surveillance, even when BMI is only mildly elevated. Conversely, greater lower-body SAT with limited ectopic fat may indicate a relatively lower-risk phenotype, although this pattern should not be interpreted as uniformly protective.

### 5.3 Emerging non-classical outcomes: mental health and beyond

The link between adipose tissue distribution and mental health is gaining attention, but the evidence is still less mature than that for cardiometabolic disease. Higher VAT has been associated with depressive symptoms and depression risk, potentially through inflammation, altered adipokine signaling, lifestyle factors, and bidirectional metabolic–psychological interactions (27, 188, 189). Large population-based studies, including analyses from the UK Biobank, also support associations between excess visceral or total adiposity and depressive symptoms, although causality remains unclear (190).

Compared with cardiometabolic outcomes, this field remains largely associative. Diet, physical activity, sleep, systemic inflammation, socioeconomic status, and existing mental health conditions may all influence both fat distribution and psychological outcomes (191, 192). Thus, VAT should not yet be treated as a direct causal driver of depression. A more balanced interpretation is that adipose distribution may form part of a broader metabolic-inflammatory phenotype that overlaps with mental health vulnerability. Longitudinal, mechanistic, and interventional studies are still needed before depot-specific adiposity can be used in mental health risk prediction.

### 5.4 Translating adipose distribution into clinical risk stratification

Depot-specific adiposity is most useful clinically when it refines, rather than replaces, conventional obesity assessment. BMI and waist-based indices remain practical entry points, but they cannot fully explain why individuals with similar body weight may differ markedly in insulin resistance, cardiometabolic risk, hepatic steatosis, or muscle quality. The added value of adipose distribution lies in identifying whether excess fat is stored in relatively safe compartments or has shifted toward VAT and ectopic sites, where metabolic risk is generally higher (4, 18).

A clinically meaningful risk model should therefore focus less on measuring every depot in isolation and more on recognizing harmful distribution patterns. Central obesity, high VAT burden, hepatic fat accumulation, pancreatic fat, and skeletal muscle lipid infiltration may indicate a metabolically high-risk phenotype that warrants closer surveillance and more intensive intervention. By contrast, greater lower-body SAT with limited ectopic fat may suggest a relatively lower-risk profile, although this pattern is not uniformly protective and should be interpreted alongside age, sex, ethnicity, lifestyle, and metabolic status.

This approach links adipose biology to practical risk stratification. In routine care, simple anthropometric measures can identify individuals who need further metabolic evaluation. In high-risk patients or research settings, imaging and functional phenotyping can help clarify whether obesity is mainly weight-related or accompanied by metabolically harmful fat redistribution. Overall, adipose tissue distribution should be used to move clinical assessment from a weight-centered model toward a depot-informed risk model (15).

Taken together, the disease relevance of adipose tissue distribution is strongest for VAT and ectopic fat in cardiometabolic disease, more context-dependent for SAT, and still emerging for mental health outcomes. The key clinical question is not whether a fat depot is simply “good” or “bad,” but whether lipid storage remains safe, whether VAT and ectopic fat have expanded, and whether depot-specific information improves risk assessment beyond BMI or total fat mass alone (193).

Depot-specific associations of adipose tissue distribution with major disease outcomes are summarized in **Table 2**.

**Table 2:** Depot-specific associations of adipose tissue distribution with major disease outcomes.

Disease category	Key adipose depot(s)	Main clinical association	Evidence type	Causality status/uncertainty	Clinical implication
Cardiovascular disease (CVD)	VAT; epicardial/pericardial fat; ectopic cardiac fat	Coronary artery disease, plaque progression, heart failure, mortality	Human imaging, epidemiology, prospective cohorts; mechanistic studies	Strong association; causal pathways partly supported	Refines cardiovascular risk beyond BMI
Type 2 diabetes mellitus (T2DM)	VAT; hepatic fat; pancreatic fat	Insulin resistance, β-cell dysfunction, glycemic deterioration	Human imaging/metabolic studies; preclinical mechanisms	Strong and biologically plausible	Identifies metabolically high-risk obesity
MASLD/organ-specific metabolic injury	Hepatic fat; VAT; skeletal muscle fat; pancreatic fat	Hepatic insulin resistance, steatosis, muscle dysfunction, β-cell stress	Human imaging/metabolic studies; preclinical feeding models	Strong for hepatic fat; muscle and pancreatic mechanisms remain more heterogeneous	Supports organ-specific risk assessment beyond BMI
Metabolic syndrome (MetS)	VAT; hepatic fat; SAT context-dependent	Central obesity, dyslipidemia, hypertension, insulin resistance	Human epidemiology and imaging	Strong for VAT; SAT effects context-dependent	Supports central adiposity-based screening
Mental health outcomes	VAT; total adiposity	Depression and depressive symptoms	Observational cohorts; emerging mechanistic data	Mostly associative; causality uncertain	Not ready for routine risk prediction

VAT, visceral adipose tissue; SAT, subcutaneous adipose tissue; T2DM, type 2 diabetes mellitus; MASLD, metabolic dysfunction-associated steatotic liver disease; MetS, metabolic syndrome.

## 6 Determinants of adipose tissue distribution

Adipose tissue distribution is shaped by a complex interplay of molecular, genetic, endocrine, and environmental factors (3). Rather than reflecting total fat mass alone, regional fat patterning depends on differences in adipocyte lineage commitment, depot-specific differentiation capacity, thermogenic potential, hormonal milieu, aging, and lifestyle-related metabolic stress (7, 194). Consequently, the relative expansion of subcutaneous, visceral, thermogenic, and ectopic fat depots varies substantially across individuals and contributes to marked heterogeneity in metabolic risk. Understanding the determinants of adipose tissue distribution is therefore essential for explaining interindividual differences in disease susceptibility and for developing more precise preventive and therapeutic strategies.

### 6.1 Molecular and genetic determinants of adipose tissue distribution

At the molecular level, adipose tissue distribution is shaped by two interconnected regulatory programs: the core adipogenic transcriptional network and depot-specific thermogenic or developmental patterning pathways (3, 195). The classical adipogenic program governs adipocyte lineage commitment and acquisition of lipid storage capacity, whereas thermogenic and developmental regulators help specify whether a given depot adopts a more subcutaneous, visceral, beige, or brown-like phenotype (22).

The core adipogenic transcriptional network is centered on PPARγ and C/EBPα, which cooperate to establish and maintain mature adipocyte identity (23). During early adipogenesis, upstream factors such as C/EBPβ and C/EBPδ initiate the differentiation program, after which PPARγ and C/EBPα drive the expression of genes involved in lipid uptake, triglyceride storage, insulin sensitivity, and adipocyte maturation (22, 196). SREBP1 further supports this process by promoting lipogenic gene expression and reinforcing adipogenic activity (63). Together, this network provides the transcriptional basis for adipocyte expansion and depot-specific fat storage capacity.

A second major regulatory axis relevant to adipose tissue distribution is the thermogenic program, particularly in subcutaneous depots with beige adipocyte potential (197). PR domain containing 16 (*PRDM16*) is a key regulator of beige and brown adipocyte identity and is closely involved in maintaining thermogenic competence in subcutaneous fat (24, 25, 198). Through interaction with peroxisome proliferator-activated receptor gamma coactivator 1α and 1β (*PGC-1α*/*PGC-1β*), *PRDM16* promotes mitochondrial biogenesis, oxidative metabolism, and thermogenic gene expression (199). The PRDM16–PGC-1α/β axis therefore helps preserve the beige-like features of subcutaneous adipose tissue and counteracts a shift toward a more visceral-like phenotype (24). When this pathway is impaired, browning capacity declines, metabolic flexibility is reduced, and adipose distribution tends to shift toward a less favorable pattern (200).

Beyond these core transcriptional programs, regional adipose patterning is further shaped by developmental regulators and signaling pathways that influence progenitor commitment, depot identity, and local adipogenic potential (26, 186). Genes such as *TBX15*, *FAM13A*, *GPC4*, and *RSPO3* have all been implicated in adipose tissue distribution. *TBX15* is linked to regional adipose identity and browning potential (201), whereas *RSPO3*, *GPC4*, and *FAM13A* appear to modulate adipose distribution through pathways related to adipocyte progenitor differentiation and WNT/β-catenin signaling (26, 202–204). These regulators help explain why abdominal and gluteofemoral depots differ in expandability, adipogenic activity, and metabolic consequences (205). In this sense, adipose tissue distribution reflects not only fat storage, but also depot-specific developmental programming.

Genetic regulation of adipose tissue distribution also shows marked sex specificity. Human studies indicate that fat patterning is more strongly sex-dependent than overall adiposity, with women tending to accumulate more lower-body SAT and men more likely to accumulate trunk and VAT (206, 207). Part of this difference appears to be mediated by local steroid metabolism. For example, aldo-keto reductase family 1 member C2 (*AKR1C2*), which participates in glucocorticoid- and androgen-related pathways, has been linked to regional fat deposition, particularly in female SAT (208, 209). In parallel, genome-wide association studies have identified several loci with sex-biased effects on fat distribution, including *LYPLAL1*, *NISCH*, and *THNSL2 (*210–212). Together, these findings suggest that the genetic regulation of adipose tissue distribution is closely intertwined with the endocrine milieu and may contribute to the distinct fat distribution phenotypes observed in men and women.

Heritability studies support a substantial genetic contribution to adipose tissue distribution. Twin and family studies indicate that body mass index has relatively high heritability, whereas WHR shows lower but still significant heritability, suggesting that regional fat patterning is also under genetic control (213, 214). Importantly, genetic influences extend beyond overall obesity susceptibility to include preferential fat deposition in specific depots and the tendency to develop trunk-dominant, gluteofemoral, or visceral phenotypes (215).

At the population level, adipose tissue distribution is further shaped by gene–environment interaction and ancestry-specific genetic architecture (28). Variants in genes such as *FTO*, *LCT*, and *FGF21* can interact with dietary patterns and nutrient composition, thereby modifying regional fat accumulation (216–218). These findings indicate that adipose tissue distribution is not a fixed genetic trait, but a dynamic phenotype shaped by both inherited susceptibility and environmental exposure (219). Ethnic differences are also biologically relevant (173). For example, Asian populations tend to accumulate more VAT at a given BMI than Europeans, whereas individuals of African ancestry generally show lower VAT and greater SAT storage capacity (220). Population-specific loci, including *NID2* and *HECTD4*, may partly contribute to these differences and help explain why metabolic risk cannot be inferred from BMI alone across ethnic groups (221).

**Figure 3:**
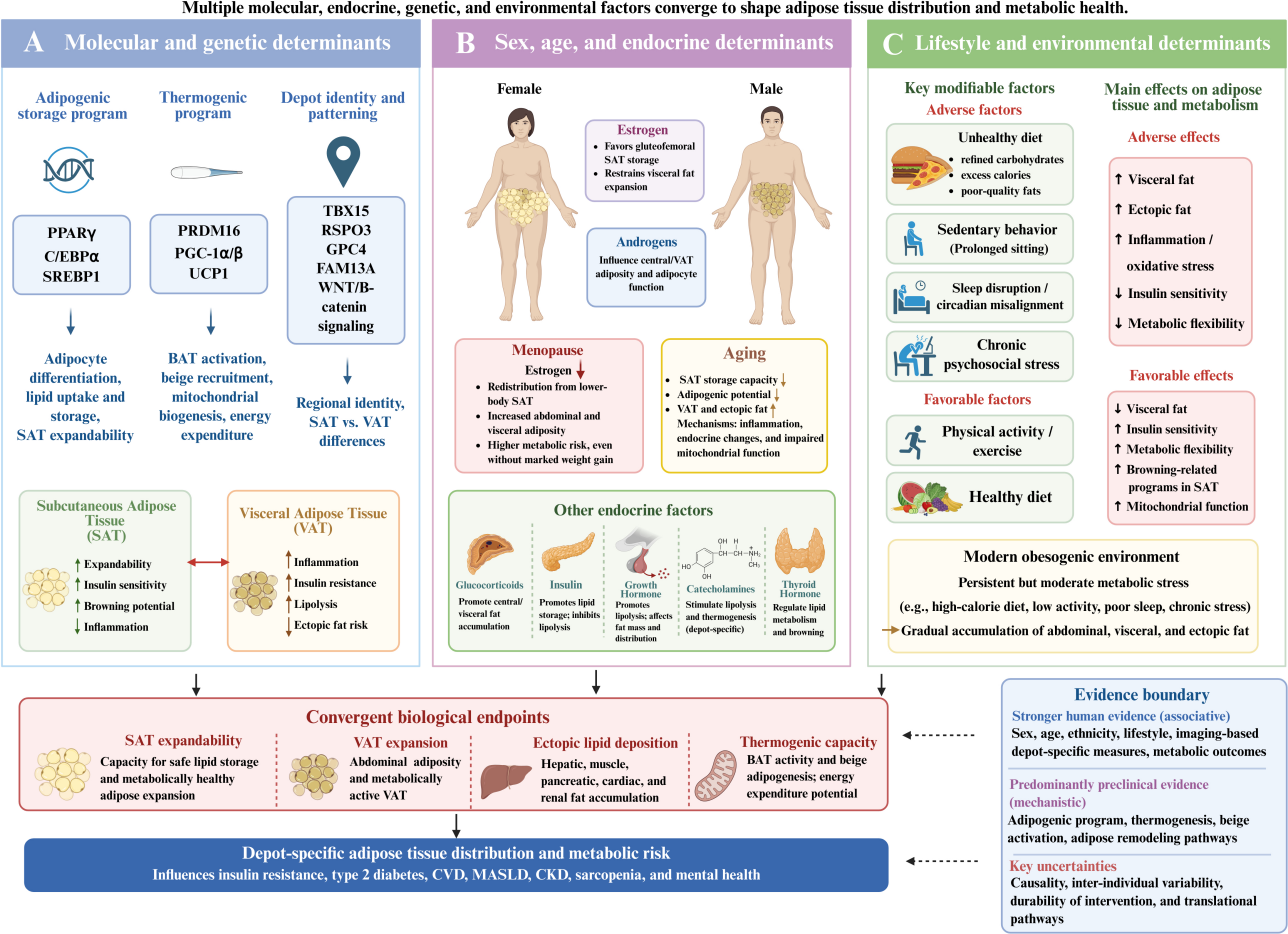
Determinants of adipose tissue distribution. **(A)** Molecular and genetic determinants. Adipose tissue distribution is regulated by coordinated molecular programs that control adipocyte differentiation, thermogenic capacity, and depot identity. The core adipogenic transcriptional network, centered on PPARγ, C/EBPα, and SREBP1, underlies adipocyte differentiation and lipid storage. In parallel, the thermogenic/beige program, including PRDM16 and PGC-1α/PGC-1β, promotes mitochondrial biogenesis, browning, and thermogenesis. Developmental and regional regulators such as TBX15, RSPO3, GPC4, FAM13A, WNT/β-catenin signaling, and AKR1C2 further shape depot-specific adipose patterning. Together, these pathways help explain the functional differences between SAT and VAT. **(B)** Sex, age, and endocrine determinants. Sex is a major determinant of regional fat patterning, with females tending to accumulate more gluteofemoral SAT and males showing greater trunk and visceral fat deposition. Estrogen generally favors lower-body SAT storage and restrains visceral fat expansion, whereas androgens influence central adiposity in a sex-dependent manner. Menopause is associated with declining estrogen levels and redistribution from lower-body SAT toward greater abdominal and visceral adiposity. Aging is accompanied by reduced SAT expandability and adipogenic capacity, together with increased VAT and ectopic fat accumulation. Additional endocrine factors, including glucocorticoids, insulin, growth hormone, catecholamines, and thyroid hormones, further modulate lipid storage, mobilization, and browning potential in a depot-specific manner. **(C)** Lifestyle and environmental determinants. Modifiable lifestyle factors substantially influence adipose tissue distribution. Unhealthy diet, sedentary behavior, sleep disruption or circadian misalignment, and chronic psychosocial stress favor visceral and ectopic fat accumulation, inflammation, oxidative stress, insulin resistance, and reduced metabolic flexibility. In contrast, regular physical activity and healthy dietary patterns are associated with lower visceral fat burden, improved insulin sensitivity, greater metabolic flexibility, and enhanced browning-related programs and mitochondrial function, particularly in SAT. Together, these molecular, endocrine, and environmental influences contribute to marked interindividual heterogeneity in adipose tissue distribution and metabolic risk, even among individuals with similar BMI.

Taken together, molecular, endocrine, genetic, and environmental determinants shape adipose tissue distribution through coordinated effects on adipogenesis, depot identity, thermogenic capacity, and lipid spillover. Still, the current evidence has clear limits. Many of the genes and pathways described above are associated with fat distribution through GWAS, depot-specific expression patterns, or preclinical models, but their causal roles in human adipose tissue are not always firmly established. In particular, direct functional validation in human SAT and VAT, longitudinal confirmation, and intervention-based evidence remain limited. These regulators are therefore best viewed as candidate mechanisms that may shape regional adipose patterning, rather than as fully proven determinants of human fat distribution. **Figure 3** summarizes this integrated framework, while **Supplementary Table 1** provides a detailed overview of representative regulators, evidence sources, translational relevance, and unresolved questions.

### 6.2 Sex, age, and endocrine determinants

Sex is one of the strongest biological determinants of adipose tissue distribution. Women generally accumulate more SAT, particularly in the gluteofemoral region, whereas men are more likely to develop upper-body and VAT accumulation (222, 223). These differences are metabolically important rather than merely anatomical, because lower-body SAT is often associated with more favorable lipid handling and lower cardiometabolic risk, whereas excess VAT is more strongly linked to insulin resistance and CVD (15, 143).

Sex hormones are central to this pattern. Estrogen tends to promote gluteofemoral and subcutaneous fat storage while limiting visceral fat expansion, whereas androgens and androgen metabolism influence both adipose distribution and adipocyte function in a sex-dependent manner (27, 207, 224, 225). Menopause provides a clear example of endocrine regulation, as declining estrogen levels are commonly accompanied by a shift from lower-body subcutaneous fat toward greater abdominal and visceral adiposity, often with worsening metabolic risk even in the absence of major weight gain (226–228).

Age is another important determinant. Over time, adipose tissue undergoes changes in expandability, cellular composition, and metabolic flexibility. With aging, the storage capacity of SAT may decline, whereas visceral and ectopic fat depots tend to expand (229). This redistribution likely reflects reduced adipogenic capacity, altered endocrine signaling, chronic low-grade inflammation, and impaired mitochondrial function, and helps explain why older adults often show a more metabolically unfavorable fat pattern even without severe obesity (230–232).

Endocrine factors beyond sex steroids also contribute (233). Glucocorticoids favor central fat accumulation and are closely linked to trunk-dominant and visceral obesity, whereas insulin, growth hormone, catecholamines, and thyroid hormones can each influence lipid storage, mobilization, and browning potential in a depot-specific manner (109, 234–237). Together, these hormonal influences interact with genetic predisposition and environmental exposure, reinforcing the dynamic and multidimensional nature of adipose tissue distribution.

### 6.3 Lifestyle and environmental determinants

Lifestyle factors substantially influence adipose tissue distribution, often beyond their effects on total body weight (238). Diet composition, habitual physical activity, sedentary behavior, sleep, and chronic psychosocial stress all affect where excess energy is stored and how different adipose depots respond to metabolic demand (28, 239–241).

Diet is particularly important because different macronutrient patterns may favor distinct adipose phenotypes. Diets high in refined carbohydrates, excess calories, or poor-quality fats tend to promote visceral and ectopic fat accumulation, whereas healthier dietary patterns may attenuate this tendency (242). These effects are further modified by gene–diet interactions, so the metabolic consequences of a given diet are not uniform across individuals.

Physical activity is another major modifier. Regular exercise is consistently associated with lower VAT burden, improved insulin sensitivity, and greater metabolic flexibility (27, 243). It may also promote browning-related programs in SAT and improve mitochondrial function, thereby shifting adipose biology toward a more favorable phenotype even when overall weight loss is modest (244, 245). By contrast, prolonged sedentary behavior is more strongly linked to central fat accumulation and reduced metabolic resilience (246).

Sleep disruption, circadian misalignment, and chronic stress may also promote adverse fat redistribution, partly through neuroendocrine and inflammatory pathways (247, 248). These influences are likely to be especially relevant in modern obesogenic environments, where persistent but moderate metabolic stress can gradually favor abdominal, visceral, and ectopic fat deposition (249). Taken together, adipose tissue distribution reflects a multilayered interaction among molecular programs, inherited susceptibility, endocrine regulation, and environmental exposure. This complexity helps explain why individuals with similar BMI can show markedly different fat distribution patterns and metabolic risk profiles, and highlights the need for more precise prevention and intervention strategies (250).

## 7 Therapeutic remodeling of adipose distribution: weight loss, redistribution, and thermogenic activation

Adipose tissue distribution is modifiable, but interventions do not remodel fat depots in the same way (251). Some mainly reduce total adiposity and secondarily decrease VAT and ectopic fat, whereas others shift lipid partitioning, improve adipose tissue function, or activate thermogenic programs (2). This distinction is important because weight loss, VAT reduction, ectopic fat reduction, lipid repartitioning, and BAT/beige activation are related but not interchangeable outcomes (29). Therapeutic effects on fat distribution should therefore be interpreted by their dominant mode of action, rather than by simply labeling interventions as lifestyle-based or pharmacological (252–255).

### 7.1 Weight-loss-dominant interventions

Weight-loss-dominant interventions reduce adipose burden mainly by creating a negative energy balance or suppressing energy intake. Their most consistent depot-related effects are reductions in VAT and ectopic fat, especially hepatic fat. However, these changes usually occur alongside overall fat loss and should not automatically be interpreted as true depot-selective redistribution.

Dietary intervention remains a practical first-line strategy. Mediterranean-style diets, green Mediterranean diets, polyphenol-enriched dietary patterns, and low-glycemic-load diets have been associated with lower central adiposity, reduced hepatic fat, smaller waist circumference, and improved metabolic risk profiles, particularly in selected populations (256–263). These benefits likely reflect better energy balance, improved diet quality, reduced inflammatory exposure, and favorable changes in hepatic lipid metabolism. Still, current evidence does not consistently show that diet alone can durably redirect lipid storage from harmful depots toward protective SAT in a depot-specific manner (264).

Glucagon-like peptide-1 receptor agonists (GLP-1RAs) provide stronger pharmacological evidence for reducing unfavorable fat accumulation. Meta-analyses show that GLP-1RAs reduce VAT and liver fat, often in parallel with substantial body weight loss (265, 266). They are therefore clinically important for improving adverse adiposity patterns. Even so, most depot-related benefits appear to track with overall fat loss. GLP-1RAs are better viewed as weight-loss-dominant therapies with favorable secondary effects on VAT and ectopic fat, rather than as classic fat-redistribution agents (252).

Bariatric surgery, if included, also fits mainly within this category. It induces large and sustained weight loss and can markedly improve VAT burden, hepatic fat, insulin resistance, and cardiometabolic risk (267, 268). However, these depot-related changes should generally be interpreted in the context of profound total fat loss and systemic metabolic remodeling, rather than as isolated depot-selective redistribution (269).

### 7.2 Redistribution-dominant interventions

Thiazolidinediones (TZDs) are the clearest example of redistribution-dominant therapy. Unlike conventional weight-loss treatments, TZDs may increase body weight while improving insulin sensitivity and shifting lipid storage away from VAT and ectopic sites toward SAT (253, 254, 270, 271). This pattern supports an important concept: metabolically beneficial adipose remodeling does not always require weight reduction.

The main value of TZDs lies in their ability to improve lipid partitioning and adipose storage capacity. Through activation of PPARγ-dependent adipogenic programs, TZDs promote the formation of smaller, more insulin-sensitive adipocytes and may enhance the capacity of SAT to store surplus lipids more safely. In this sense, TZDs are better viewed as lipid-repartitioning agents than as anti-obesity drugs (254, 272, 273). However, their broader use is limited by adverse effects, including weight gain, fluid retention, edema, and other safety concerns (274, 275). Thus, TZDs provide a useful proof of principle for therapeutic fat redistribution, but they are not an ideal general strategy for obesity management.

### 7.3 Thermogenic or browning-directed interventions

Thermogenic strategies aim to increase energy expenditure and metabolic flexibility by activating BAT or recruiting beige adipocytes within WAT. Cold exposure is the most physiological approach. Human studies and recent reviews indicate that repeated cold exposure can increase BAT activity and energy expenditure, and may shift adipose biology toward a more thermogenic phenotype (276–279). However, the present clinical evidence is not strong enough to treat cold exposure as a standard therapy for adverse fat distribution. It is better described as an emerging adjunct that targets thermogenic capacity rather than a validated method for durable redistribution of body fat (276).

Several additional pharmacological strategies are promising but remain less established. β3-adrenergic agonists, particularly mirabegron, can activate human BAT and increase resting energy expenditure, with some evidence of improved high-density lipoprotein cholesterol and insulin sensitivity (31, 32). Still, whether these effects translate into sustained fat loss or durable depot remodeling is unresolved. Other browning-related approaches, such as fibrates and PPARα agonists, have shown thermogenic or beige-fat-related effects mainly in preclinical models, while human evidence remains limited (280, 281).

Overall, thermogenic and browning-directed interventions are attractive because they target energy expenditure and WAT plasticity rather than appetite or body weight alone. At present, however, they are better viewed as promising but incompletely validated strategies for adipose tissue remodeling.

### 7.4 Metabolic-function-dominant interventions

Some interventions improve adipose distribution and metabolic health mainly by enhancing tissue function rather than directly redistributing fat. Exercise is the clearest example. Aerobic training, resistance training, interval-based exercise, and combined programs can reduce VAT, improve insulin sensitivity, preserve or increase lean mass, and enhance metabolic flexibility (244, 282–284). These benefits may occur even with modest weight loss, suggesting that exercise acts not only as a weight-loss strategy but also as a functional therapy for adipose and muscle tissue.

Metformin also fits better into this category than into a true redistribution model. It has modest effects on body weight and waist circumference (WC), and some studies suggest VAT reduction in selected populations (285, 286). However, evidence for durable depot-specific redistribution remains limited. Its main value lies in improving insulin sensitivity, hepatic glucose metabolism, and overall metabolic function rather than selectively shifting lipid storage from VAT to SAT (30).

Diet quality can be interpreted in a similar way. Beyond caloric restriction, healthy dietary patterns may improve inflammation, insulin sensitivity, hepatic lipid metabolism, and cardiometabolic risk (256–263). These changes may indirectly reduce VAT and ectopic fat burden, but they should not be taken as direct evidence of true fat redistribution.

Other pharmacological strategies remain more exploratory. Cannabinoid receptor type 1 (CB1) pathway modulation may affect visceral fat biology and insulin sensitivity, but centrally acting CB1 antagonists such as rimonabant were limited by psychiatric adverse effects, and peripheral approaches remain investigational (287–290). Likewise, setmelanotide is effective in selected monogenic obesity syndromes through melanocortin 4 receptor (MC4R) signaling, but its main effect is appetite and body weight reduction rather than proven depot-selective remodeling (291).

### 7.5 Overall perspective

Taken together, current interventions should not be treated as interchangeable approaches to fat redistribution. Lifestyle intervention, GLP-1 receptor agonists, and bariatric surgery mainly reduce total adiposity, with secondary improvements in VAT and ectopic fat. Thiazolidinediones (TZDs) provide the clearest clinical example of lipid repartitioning toward SAT, although their use is limited by adverse effects. Exercise and metformin primarily improve metabolic function, whereas thermogenic strategies remain biologically promising but are not yet established for durable fat redistribution.

The key therapeutic question is therefore not simply whether an intervention reduces body weight, but whether it improves the pattern of fat storage and metabolic risk. Future trials should include depot-specific endpoints, such as VAT volume, SAT distribution, hepatic fat, skeletal muscle lipid infiltration, BAT activity, and markers of adipose inflammation or fibrosis. These outcomes would help distinguish weight-loss-driven improvement from genuine remodeling of adipose distribution and may support more precise, mechanism-based obesity treatment. To clarify these distinctions, **Supplementary Table 2** summarizes current interventions according to their dominant mode of action, depot-related effects, evidence for true fat redistribution, dependence on weight loss, and remaining clinical uncertainties.

## 8 Discussion and future perspectives

### 8.1 Conceptual advance: from fat quantity to depot function

Adipose tissue distribution offers a more informative framework for understanding obesity-related risk than total fat mass or BMI alone. The key point is that fat distribution is not just an anatomical map of lipid storage. It reflects the balance between safe lipid storage, depot expandability, lipid spillover, thermogenic capacity, and pathological remodeling. Accordingly, the clinical question shifts from simply “how much fat is present” to “where fat is stored, whether adipose tissue remains functional, and whether lipid excess has reached metabolically vulnerable organs.” (3, 15, 29).

This view helps explain why individuals with similar BMI can have very different metabolic phenotypes. Expandable SAT, especially lower-body SAT, can buffer surplus lipids when adipogenesis, vascular adaptation, extracellular matrix flexibility, and immune homeostasis are preserved. In contrast, limited SAT expandability, excessive VAT expansion, and ectopic lipid deposition in the liver, skeletal muscle, pancreas, heart, and kidney indicate a shift from adaptive storage to lipid spillover and organ-specific metabolic stress. Thermogenic adipose tissues, including BAT and beige adipocytes, add another functional layer by regulating energy expenditure, substrate oxidation, and white adipose tissue plasticity (13, 100).

Thus, the value of this review is not simply to describe individual fat depots, but to connect depot-specific biology with clinical assessment and therapeutic remodeling. SAT, VAT, thermogenic fat, and ectopic lipid deposition are interconnected parts of a metabolic system shaped by storage capacity, inflammatory remodeling, endocrine regulation, lifestyle exposure, genetic background, and treatment response. This integrated view supports a shift from weight-centered obesity assessment toward depot-informed precision medicine.

### 8.2 Evidence hierarchy: what is established, probable, and uncertain

The evidence for depot-specific adiposity is uneven across tissues, diseases, and interventions. The strongest evidence concerns VAT and ectopic fat in cardiometabolic disease. Human imaging, epidemiological, and prospective studies consistently link excess VAT, hepatic fat, cardiac fat, and other ectopic lipid depots with insulin resistance, type 2 diabetes mellitus, metabolic syndrome, cardiovascular disease, and organ-specific injury (122, 164). These associations are biologically plausible, as VAT and ectopic fat are closely related to increased free fatty acid flux, chronic low-grade inflammation, adipokine dysregulation, impaired insulin signaling, and tissue-specific lipotoxicity (2, 14, 15).

A second group of evidence is strong but more context-dependent. SAT, especially gluteofemoral SAT, is often associated with a more favorable metabolic profile, but this protection is not absolute. Its effect depends on anatomical location, expansion pattern, adipocyte size, inflammatory status, vascularization, and extracellular matrix flexibility. Lower-body SAT may act as a relatively safe lipid reservoir, whereas abdominal SAT or maladaptively expanded SAT may contribute to lipid spillover and metabolic dysfunction (12, 51–54). Likewise, BAT activity and beige adipocyte recruitment are associated with favorable metabolic profiles and improved substrate utilization, but whether these effects translate into durable fat redistribution in humans remains uncertain (13, 31, 32).

More emerging areas include non-classical outcomes, long-term thermogenic interventions, and omics-based clinical translation. VAT and total adiposity have been associated with depressive symptoms and other mental health outcomes, but these links remain largely associative and may be influenced by inflammation, sleep, diet, physical activity, socioeconomic status, and bidirectional metabolic–psychological interactions (165, 188–190). Similarly, many molecular and cellular mechanisms of adipose depot remodeling are still supported mainly by animal models or ex vivo studies. These data are valuable for understanding causality, but translation to humans needs caution because murine and human adipose depots are not fully equivalent, particularly for VAT and inducible thermogenic fat (33, 83).

This evidence hierarchy should guide how depot-related findings are interpreted. VAT and ectopic fat are clinically informative features of metabolically harmful adiposity, but they should not be treated as stand-alone causal explanations for all obesity-related complications. BAT activation, beige adipocyte recruitment, and molecular remodeling pathways are promising therapeutic directions, but their durability, safety, and real-world effect size remain uncertain. In practical terms, some depot-related findings are ready to inform risk stratification, whereas others are better viewed as mechanistic hypotheses or early translational opportunities.

### 8.3 Translational framework for precision obesity medicine

A depot-informed framework may improve obesity risk stratification by moving beyond BMI-centered classification. BMI and waist-based indices remain useful for screening, but they cannot fully capture fat location, adipose tissue function, or ectopic lipid deposition (4, 18). More precise phenotyping, including imaging-based assessment of SAT, VAT, hepatic fat, skeletal muscle lipid infiltration, and thermogenic activity, can help identify harmful distribution patterns that are missed by body weight alone (16–21, 160–162). The clinical value of this approach lies in earlier recognition of harmful fat distribution before overt metabolic disease develops. Individuals with visceral obesity, hepatic fat accumulation, or early ectopic lipid deposition may already carry increased metabolic risk even when BMI is only mildly elevated. Identifying these patterns at an earlier stage could support timely lifestyle intervention, closer metabolic monitoring, and, when appropriate, more intensive pharmacological or surgical strategies. In this sense, adipose distribution is not only a descriptive phenotype, but also a potential tool for preventing the progression from obesity to insulin resistance, type 2 diabetes mellitus, metabolic dysfunction–associated steatotic liver disease, and cardiovascular disease.

Clinically, the goal is not to measure every fat depot in every patient, but to recognize patterns that meaningfully change risk assessment and management. Central obesity, high VAT burden, hepatic steatosis, pancreatic fat, skeletal muscle lipid infiltration, and poor muscle quality may indicate a metabolically high-risk phenotype requiring closer surveillance or more intensive intervention. In contrast, greater lower-body SAT with limited ectopic fat may suggest a relatively lower-risk pattern, although this should always be interpreted alongside age, sex, ethnicity, menopause status, lifestyle, and metabolic biomarkers.

This framework also helps interpret therapeutic response. Lifestyle intervention, GLP-1 receptor agonists, and bariatric surgery mainly reduce total adiposity with secondary improvements in VAT and ectopic fat, whereas thiazolidinediones provide the clearest example of lipid repartitioning toward SAT. Exercise and metformin primarily improve metabolic function, while thermogenic strategies remain promising but not yet established for durable fat redistribution. Future treatment evaluation should therefore distinguish weight-loss-driven improvement from genuine remodeling of adipose distribution.

### 8.4 Future directions

Future research should make depot-specific phenotyping more standardized and clinically usable. At present, no available method fully meets this need. Anthropometric indices are easy to apply and useful for initial screening, but they cannot separate SAT, VAT, and ectopic fat. CT and MRI offer more precise depot-level assessment, although cost, availability, CT-related radiation exposure, and the lack of agreed clinical thresholds still limit their broader use. Functional approaches and omics-based profiling add information on depot activity and molecular remodeling, but they are still mainly research tools. Moving forward, the field needs harmonized definitions, reproducible protocols, and practical risk cut-offs so that adipose distribution can be used not only for description, but also for early detection, risk stratification, and timely intervention.

A second priority is to build stronger human-centered mechanistic evidence. Current studies have identified several regulatory layers that may shape adipose distribution, including adipogenic transcriptional programs, thermogenic pathways, developmental patterning, sex hormone signaling, genetic susceptibility, inflammation, extracellular matrix remodeling, and gene–environment interactions. However, much of this evidence still comes from animal models, cell experiments, or cross-sectional human studies. How these mechanisms directly determine fat distribution in humans, and how their effects vary by sex, age, ethnicity, and metabolic status, remains insufficiently defined. Future work should combine human imaging, adipose tissue biopsy, single-cell and spatial omics, and longitudinal metabolic phenotyping to clarify which mechanisms truly drive healthy adipose expansion, maladaptive remodeling, and lipid spillover in humans.

Intervention studies should also move beyond body weight, BMI, and waist circumference (WC) as the main outcomes. Depot-specific endpoints, such as VAT volume, SAT distribution, hepatic fat, skeletal muscle lipid infiltration, BAT activity, and markers of adipose inflammation, fibrosis, and metabolic function, would help determine whether an intervention simply reduces fat mass or genuinely changes adipose distribution and tissue quality. In the long term, adipose distribution should be interpreted within a precision medicine framework that considers sex, age, menopause, ethnicity, genetic background, lifestyle, medication exposure, and baseline metabolic status. This approach may help identify high-risk individuals despite only modest BMI elevation, guide treatment according to the dominant pattern of adipose dysfunction, and move adipose distribution from a descriptive phenotype toward a practical tool for mechanism-guided obesity management.

## 9 Conclusion

Adipose tissue distribution is a key determinant of metabolic risk and should be considered beyond total fat mass or BMI alone. Different adipose depots, including SAT, VAT, thermogenic fat, and ectopic lipid stores, have distinct biological properties and disease relevance. Excess VAT and ectopic fat are most consistently linked to cardiometabolic disease, whereas lower-body SAT and thermogenic adipose tissue may exert more favorable metabolic effects under specific conditions. Current advances in imaging, functional metabolic assessment, and omics-based approaches have improved depot-specific phenotyping, but standardized definitions, human-centered mechanistic studies, and longitudinal clinical validation remain insufficient. Future research should distinguish weight-loss–driven metabolic improvement from genuine adipose redistribution and should integrate depot-specific imaging, functional assessment, genetic background, lifestyle exposure, and therapeutic response. Such an integrated framework may support more precise risk stratification and guide targeted interventions for obesity-related metabolic disease.
