# Supplementary material for: Adipose tissue distribution in metabolic disease: depot-specific biology, clinical assessment, and therapeutic remodeling
Source: Front Endocrinol (Lausanne). 2026 Jun 24;17:1822659. doi: 10.3389/fendo.2026.1822659 (PMC13341497; doi:10.3389/fendo.2026.1822659)
Supplement: Supplementary file 1 [file Table1.docx]

**Supplementary Table**

**Supplementary Table S1. Mechanistic determinants of adipose distribution and their translational evidence.**

| **Mechanistic axis** | **Representative regulators** | **Main depot effect** | **Evidence source** | **Human relevance** | **Remaining uncertainty** |
| --- | --- | --- | --- | --- | --- |
| Core adipogenic storage program | PPARγ, C/EBPα, C/EBPβ/δ, SREBP1 | Promote adipocyte differentiation, lipid uptake, and triglyceride storage capacity in SAT and VAT | Cell and animal studies; human adipose tissue studies | Central to adipose expandability and safe lipid storage | Depot-specific regulation in human SAT versus VAT remains incompletely defined |
| Adipose expandability and healthy remodeling | PPARγ pathway, angiogenic factors, ECM remodeling programs | Support hyperplastic expansion, smaller adipocytes, adequate vascularization, and flexible ECM remodeling | Human obesity studies; preclinical models | Help explain why some individuals store excess lipids safely while others develop VAT and ectopic fat accumulation | Reliable clinical markers of “safe” versus maladaptive expansion are still lacking |
| Pathological hypertrophy, inflammation, and fibrosis | TNF-α, IL-6, macrophage-related pathways, collagens, fibronectin, TGF-β-related ECM programs | Promote adipocyte hypertrophy, hypoxia, immune infiltration, fibrosis, and reduced lipid-buffering capacity | Human adipose biopsies; animal models | Link adipose dysfunction to insulin resistance and metabolic disease | Causal sequence among hypertrophy, inflammation, fibrosis, and lipid spillover remains difficult to resolve in humans |
| Thermogenic and beige adipocyte program | PRDM16, PGC-1α/PGC-1β, UCP1 | Promote BAT activity, beige adipocyte recruitment, mitochondrial biogenesis, and energy expenditure | Strong preclinical evidence; human BAT imaging and metabolic studies | Provide a mechanistic basis for thermogenic fat as a metabolic modifier | Long-term clinical impact of BAT/beige activation on durable fat redistribution remains uncertain |
| Depot identity and developmental patterning | TBX15, GPC4 | Contribute to regional differences between abdominal, gluteofemoral, subcutaneous, and visceral depots | Human adipose gene-expression studies; developmental and experimental models | Helps explain depot-specific adipogenic capacity and metabolic behavior | Developmental markers are not yet directly usable for clinical risk stratification |
| WNT-related progenitor regulation | WNT/β-catenin signaling, RSPO3 | Regulate adipocyte progenitor commitment and regional adipogenesis | GWAS, human adipose progenitor studies, experimental models | Relevant to abdominal versus gluteofemoral fat distribution and SAT expandability | Effects may vary by depot, sex, ancestry, and metabolic state |
| Genetic susceptibility to fat distribution | FAM13A, FTO, LYPLAL1, NISCH, THNSL2 | Influence central adiposity, WHR, trunk versus lower-body fat patterning, and ectopic fat tendency | GWAS, Mendelian randomization, population studies | Support the genetic contribution to fat distribution beyond BMI | Most loci have small effects, and biological mechanisms remain partly unresolved |
| Sex-specific and endocrine regulation | Estrogen signaling, androgen signaling, AKR1C2, glucocorticoid-related pathways | Shape female-predominant lower-body SAT storage, male-predominant central/VAT accumulation, and menopause-related fat redistribution | Human epidemiology, endocrine studies, animal models | Highly relevant for sex differences, menopause, and individualized metabolic risk | Interaction between sex hormones, local steroid metabolism, and depot-specific adipose biology remains complex |
| Gene–environment interaction | FTO, LCT, FGF21, diet-responsive metabolic pathways | Modify regional fat accumulation in response to diet, nutrient composition, and lifestyle exposure | Human dietary intervention trials; genetic association studies | Explains why individuals with similar BMI or diet may show different fat distribution patterns | Findings are population- and context-dependent; clinical prediction remains limited |
| Ectopic lipid spillover pathways | Adipose lipolysis, hepatic de novo lipogenesis, mitochondrial oxidative capacity, inflammatory pathways | Promotes lipid accumulation in liver, skeletal muscle, pancreas, heart, and kidney | Human imaging/metabolic studies; preclinical feeding models | Directly links impaired adipose buffering to organ-specific metabolic injury | Distinguishing cause from consequence remains challenging, especially in longitudinal human studies |

**Abbreviations:** BAT, brown adipose tissue; C/EBP, CCAAT/enhancer-binding protein; ECM, extracellular matrix; GWAS, genome-wide association study; PGC-1α/β, peroxisome proliferator-activated receptor gamma coactivator 1α/β; PPARγ, peroxisome proliferator-activated receptor γ; SAT, subcutaneous adipose tissue; TGF-β, transforming growth factor-β; UCP1, uncoupling protein 1; VAT, visceral adipose tissue; WHR, waist-to-hip ratio.

**Supplementary Table S2. Therapeutic strategies targeting adipose distribution and depot-specific metabolic remodeling**

| **Intervention category** | **Representative examples** | **Dominant mode of action** | **Main depot-related effects** | **Evidence for true fat redistribution** | **Weight-loss dependence** | **Clinical maturity / key uncertainty** |
| --- | --- | --- | --- | --- | --- | --- |
| Dietary intervention | Mediterranean diet, green Mediterranean diet, low-glycemic-load diet, polyphenol-enriched diet | Weight-loss-dominant / metabolic-function-dominant | Reduces central adiposity and hepatic fat; improves inflammatory and metabolic profiles | Limited; mainly improves VAT and ectopic fat alongside overall metabolic improvement | Moderate to high | Established first-line strategy; durable depot-selective redistribution remains unproven |
| GLP-1 receptor agonists | Liraglutide, semaglutide | Weight-loss-dominant | Reduces body weight, VAT, hepatic fat, and adverse adiposity patterns | Limited; depot effects largely track with overall fat loss | High | Established for obesity and metabolic disease; not a classic redistribution therapy |
| Bariatric surgery | Sleeve gastrectomy, Roux-en-Y gastric bypass | Weight-loss-dominant / systemic metabolic remodeling | Markedly reduces total fat mass, VAT, hepatic fat, and insulin resistance | Limited as isolated redistribution; depot changes occur with profound total fat loss | High | Established for severe obesity; depot-specific remodeling should be interpreted in relation to overall weight loss |
| Thiazolidinediones | Pioglitazone, rosiglitazone | Redistribution-dominant | Shifts lipid storage away from VAT and ectopic sites toward SAT; improves insulin sensitivity | Strongest clinical signal for lipid repartitioning | Low or paradoxical, because body weight may increase | Established for selected metabolic indications; limited by weight gain, edema, fluid retention, and safety concerns |
| Cold exposure | Repeated mild cold exposure, cold acclimation | Thermogenic activation | Activates BAT, increases nonshivering thermogenesis, and may promote a more thermogenic adipose phenotype | Uncertain; evidence for durable fat redistribution is limited | Low to moderate | Emerging adjunct; feasibility, adherence, durability, and clinical magnitude remain uncertain |
| β3-adrenergic agonists | Mirabegron | Thermogenic activation | Activates human BAT, increases resting energy expenditure, and may improve HDL cholesterol and insulin sensitivity | Uncertain; sustained depot remodeling has not been established | Low to moderate | Promising but not established for obesity treatment or durable adipose redistribution |
| PPARα agonists / fibrates | Fenofibrate and related agents | Browning-related / lipid-metabolic modulation | May promote thermogenic or beige-fat-related programs, mainly in preclinical models | Weak in humans | Unclear | Human evidence for fat redistribution remains limited; mainly mechanistic or preclinical support |
| Exercise | Aerobic training, resistance training, HIIT, combined exercise | Metabolic-function-dominant | Reduces VAT, improves insulin sensitivity, preserves or increases lean mass, and enhances metabolic flexibility | Limited as true redistribution; improves tissue function and VAT burden | Low to moderate | Established lifestyle therapy; benefits may occur even with modest weight loss |
| Metformin | Metformin | Metabolic-function-dominant | Modestly reduces body weight and WC; may reduce VAT in selected populations; improves insulin sensitivity and hepatic glucose metabolism | Weak; durable depot-specific redistribution is not established | Low | Established metabolic drug; not primarily a fat-redistribution therapy |
| CB1 pathway modulators | Rimonabant, peripheral CB1 antagonists | Exploratory metabolic modulation | May affect visceral fat biology and insulin sensitivity | Unclear | Variable | Centrally acting agents limited by psychiatric adverse effects; peripheral approaches remain investigational |
| MC4R agonists | Setmelanotide | Appetite / body-weight regulation in monogenic obesity | Reduces appetite and body weight in selected monogenic obesity syndromes | Not proven | High | Established for rare genetic obesity; not a depot-selective remodeling therapy |

**Abbreviations:** BAT, brown adipose tissue; CB1, cannabinoid receptor type 1; GLP-1, glucagon-like peptide-1; HDL, high-density lipoprotein; HIIT, high-intensity interval training; MC4R, melanocortin 4 receptor; PPARα, peroxisome proliferator-activated receptor α; SAT, subcutaneous adipose tissue; VAT, visceral adipose tissue; WC, waist circumference.
